# Hopelessness and externality as predictors of experiencing anger during COVID-19 lockdown in Russia

**DOI:** 10.1192/j.eurpsy.2021.757

**Published:** 2021-08-13

**Authors:** T. Gordeeva, O. Sychev, M. Dovger

**Affiliations:** 1 Psychology, Lomonosov Moscow State University, Moscow, Russian Federation; 2 Research Department, Shukshin Altai State University for Humanities and Pedagogy, Moscow, Russian Federation

**Keywords:** anger, COVID-19 lockdown, Hopelessness, externality

## Abstract

**Introduction:**

Following Italy and many other European countries Russia entered a nationwide lockdown in March 2020. Since quarantine had impact on mental health (Gualano et al., 2020, Stanton et al., 2020), this study aimed to study the psychological predictors of low mental health and anger in Russian university students. Previous studies have shown that young people are most vulnerable part of population during Covid-19 pandemic (Pervichko et al., 2020).

**Objectives:**

The purpose of this research was to assess the effects of externality and hopelessness on anger and irritation during COVID-19 lockdown.

**Methods:**

The sample comprised 120 university students (86% women, M=18.84, SD=1.58) from Moscow. Online survey has been conducted in April 2020. Measures included Russian externality-internality scale based on Rotter’s scale and three new scales specific for COVID-19 pandemic developed for this study to assess feeling of hopelessness (α = 0.72), anger (α = 0.70) and positive reformulation (α = 0.84).

**Results:**

Anger shows significant correlations with hopelessness (r=0.43; p<0.001), externality (r=0.29; p<0.01) and positive reformulation (r=–0.41; p<0.001). Structural equation modeling confirms theoretical model according to which the effect of externality on anger is mediated by hopelessness and positive reformulation (negatively) (indirect effects sig. at p<0.01, χ2 = 1.32; df = 1; p = 0.251; CFI = 0.995; TLI = 0.969; RMSEA = 0.052.

**Conclusions:**

Conclusions. Anger and irritation regarding the necessity to stay at home during COVID-19 lockdown may be caused by external locus of control which effect on anger is mediated by hopelessness and limited capacities for positive reframing.

